# Beyond the stomach: the association between *Helicobacter pylori* and the spectrum of digestive cancers

**DOI:** 10.3389/fcimb.2025.1633227

**Published:** 2025-09-02

**Authors:** He Xin, Chi Zhu, Chan Zhu, Xing Zhang, Dongsheng Chen, Qingping Wang

**Affiliations:** ^1^ Department of Radiology, Shengjing Hospital of China Medical University, Shenyang, Liaoning, China; ^2^ Intervention Ward One, Shengjing Hospital of China Medical University, Shenyang, Liaoning, China; ^3^ The State Key Laboratory of Neurology and Oncology Drug Development, Jiangsu Simcere Diagnostics Co., Ltd, Nanjing, China; ^4^ Department of Medicine, Nanjing Simcere Medical Laboratory Science Co., Ltd., Nanjing, China; ^5^ Tianjin Second People’s Hospital, Tianjin, China; ^6^ Tianjin Institute of Hepatology, Tianjin, China

**Keywords:** *Helicobacter pylori*, hepatocellular carcinoma, cholangiocarcinoma, colorectal cancer, esophageal carcinoma

## Abstract

*Helicobacter pylori* (*H. pylori*) is a Group 1 gastric carcinogen increasingly implicated in extragastric digestive malignancies. This review synthesizes evidence on its role in liver, biliary, esophageal, colorectal, and pancreatic cancers. Based on its unique spiral morphology, flagellar motility bundle, and urease activity-mediated acidic niche adaptation, *H. pylori* disrupts host cellular homeostasis through multifactorial virulence mechanisms involving CagA/VacA synergy, and exploits antigenic variation and immunomodulatory strategies to achieve persistent gastric mucosal colonization and chronic infection. Emerging evidence suggests associations between *H. pylori* infection and nongastric digestive cancers, though relationships vary by site. For hepatocellular carcinoma (HCC), epidemiological studies indicate increased risk (OR 4.75), particularly with HCV coinfection, but mechanistic and cohort data remain conflicting. Biliary tract cancer (BTC) shows stronger epidemiological links, especially for cholangiocarcinoma (OR 4.18), supported by virulence factor detection. In esophageal cancer, *H. pylori* particularly CagA+ strains demonstrates a protective effect against adenocarcinoma but no significant association with squamous cell carcinoma. Colorectal cancer exhibits complex associations, with meta-analyses suggesting increased risk in East Asian populations and potential benefits from eradication therapy. Pancreatic cancer links remain inconsistent. Proposed mechanisms of *H. pylori* in extragastric cancers include chronic inflammation, virulence factor activity and microbiome disruption. This comprehensive review synthesizes contemporary evidence on the bacterium’s role in non-gastric digestive malignancies, examines pathways underlying its oncogenicity, and outlines translational implications for risk stratification and therapeutic innovation.

## 1 Introduction

Since Marshall and Warren’s pioneering isolation of Helicobacter pylori (*H. pylori*) from gastric mucosa in 1982 (Marshall and Warren, 1984), this spiral-shaped gram-negative bacterium has revolutionized our understanding of chronic gastritis and peptic ulcer pathogenesis. Its microaerophilic growth requirements and persistent colonization of over 40% of the global adult population have established *H. pylori* as a formidable clinical challenge (Li et al., 2023). Notably, the bacterium’s carcinogenic potential was formally recognized by the International Agency for Research on Cancer (IARC) in 1994, classifying it as a Group 1 carcinogen (Schistosomes, liver flukes and Helicobacter pylori, 1994).

A 2017 meta analysis that looked into the link between 31 infections and cancer found that around 4.3% of adult cancers were due to eight particular infections, with *H. pylori* being a significant one (Volesky-Avellaneda et al., 2023). It is well - known that *H. pylori* is connected to gastric cancer (GC), and the overall occurrence of GC in people with *H. pylori* infection was determined to be 17.4%. Interestingly, GC rates among *H. pylori* - infected individuals differ greatly from country to country, with Asian nations having the highest rates (Pormohammad et al., 2019). Such pathophysiological complexity underscores the need for region-specific prevention strategies addressing this persistent global health burden. Beyond its established gastric tropism, emerging evidence implicates *H. pylori* in extragastric oncogenesis, including hepatocellular carcinoma (HCC), cholangiocarcinoma (CCA), colorectal cancer (CRC), pancreatic malignancies, and esophageal carcinoma (EC) (de Martel et al., 2020). This expanded oncogenic spectrum appears modulated through both direct microbial interactions and systemic inflammatory cascades, with risk stratification influenced by strain-specific virulence factors, host polymorphisms, and environmental cofactors (Waldum and Fossmark, 2021). Wu et al. found that *H. pylori* increases expression of protein arginine deiminase type 4 (PAD4) by stabilizing hypoxia-inducible factor 1α (HIF-1α), worsening rheumatoid arthritis (Wu et al., 2024). Additionally, some preliminary studies have revealed a connection between *H. pylori* infection and the occurrence of nonthyroidal illness syndrome, anemia, and iron deficiency (Hou et al., 2019; Sun et al., 2021; Lee et al., 2022).

Understanding the connection between *H. pylori* and non - gastric digestive cancers is very important, as it may reveal new strategies for prevention and treatment. Knowing how *H. pylori* might contribute to these cancers could help create targeted treatments, especially in areas where *H. pylori* infection is common. Also, what we learn from this connection could help make public health strategies to reduce cancer cases around the world.

This comprehensive analysis synthesizes current evidence elucidating *H. pylori’s* emerging oncogenic potential beyond gastric malignancies, with particular focus on digestive cancers. Through systematic evaluation of molecular epidemiological data and experimental models, we delineate pathogen-host interactions driving extragastric tumorigenesis while critically assessing translational implications for early detection and therapeutic intervention.

## 2 Biological properties of H. pylori

### 2.1 Morphological and physiological features of H. pylori

The spiral morphology and polar flagellar bundle of *H. pylori* confer exceptional motility through viscous mucus, enabling strategic positioning at the epithelial interface where pH gradients stabilize (Marshall and Warren, 1984). This ecological specialization is augmented by urease-mediated microenvironment remodeling - enzymatic conversion of urea generates localized ammonia buffers that transiently elevate peri-bacterial pH to 6.0-7.0, facilitating survival despite luminal acidity (Weeks et al., 2000; Malfertheiner et al., 2023). Genomic plasticity further enhances ecological persistence, with phase-variable expression of outer membrane proteins creating antigenic mosaicism that confounds immune surveillance (Suerbaum and Michetti, 2002).

### 2.2 Pathogenic mechanisms of *H. pylori*

The pathogenicity of *H. pylori* is multifactorial and involves a complex interplay of bacterial factors and host responses. Its pathogenicity is mainly due to its virulence factors, such as urease, adhesins, toxins, and secretion systems (Suerbaum and Michetti, 2002). Urease is capable of hydrolyzing urea to produce ammonia and carbon dioxide, neutralizing gastric acid and providing an optimal pH environment for *H. pylori* survival (Debowski et al., 2017). The bacterium secretes a variety of virulence factors, including cytotoxin-associated gene A (CagA) and vacuolating cytotoxin A (VacA), which play a key role in causing gastric inflammation and epithelial cell damage (de Brito et al., 2019). The Cag pathogenicity island-encoded T4SS injects phosphorylatable CagA into parietal cells, inducing cytoskeletal rearrangement via SHP-2/Rho GTPase pathways while activating β-catenin-mediated proliferative signaling (Hatakeyama, 2004; Backert et al., 2015). VacA exhibits pleiotropic toxicity, with inducing acidic vacuolation in host cell and its pore-forming capacity disrupting mitochondrial membrane potentials to inhibit apoptosis, while simultaneously blocking T-cell activation through CD25/LFA-1 interference (Ansari and Yamaoka, 2020; Baj et al., 2020). Recent single-cell transcriptomic studies reveal these virulence factors synergistically induce epithelial-mesenchymal transition (EMT) signatures and cancer stem cell marker expression in preneoplastic lesions (Zhu et al., 2017).By catalyzing the hydrolysis of glutamine and glutathione, γ-glutamyl-transpeptidase (GGT) facilitates the generation of ROS and ammonia. This process induces cell cycle arrest, apoptosis, and necrosis while concurrently suppressing T-cell proliferation and dendritic cell differentiation, consequently promoting *H. pylori*-associated ulceration and inflammation (Roche et al., 2004). The outer membrane proteins of *H. pylori* that bind to host epithelial cell receptors, such as OipA, BabA, and SabA, promote bacterial adhesion and induce chronic inflammation through IL-8 secretion (Duan et al., 2025) (**Table 1**).

**Table 1:** Pathogenic mechanisms of *H. pylori*.

Virulence factors	Pathogenic mechanisms	References
Urease	Converts urea to NH_3_/CO_2_, neutralizing acid environment to favor H. pylori survival	(Roche et al., 2004)
CagA	Translocates into epithelial cells by T4SS, disrupts the cytoskeleton, and triggers proliferative signaling cascades	(Hatakeyama, 2004; Backert et al., 2015)
VacA	Vacuolates of host cells, affects mitochondria to induce apoptosis, and inhibits of T-cell activation	(Ansari and Yamaoka, 2020; Baj et al., 2020)
GGT	Generates ROS and ammonia to induce cell-cycle arrest, apoptosis, and necrosis while suppressing T-cell proliferation and dendritic-cell differentiation	(Shibayama et al., 2007; de Brito et al., 2019)
OipA	Induces mitochondrial apoptosis cascade, resulting in cytotoxicity and gastric mucosal inflammation	(Teymournejad et al., 2017)
BabA	Specifically binds to Le^b^ blood-group antigens on gastric epithelia to mediate tight adhesion, synergizes with CagA/VacA to amplify inflammation, and promotes DNA double-strand breaks	(Roesler et al., 2014)
SabA	Mediates firm adhesion, triggers neutrophil phagocytosis, and modulates the oxidative burst, thereby driving chronic inflammation	(Roche et al., 2004; Muzaheed, 2020)

### 2.3 Immune response to H. pylori infection

**Figure 1:**
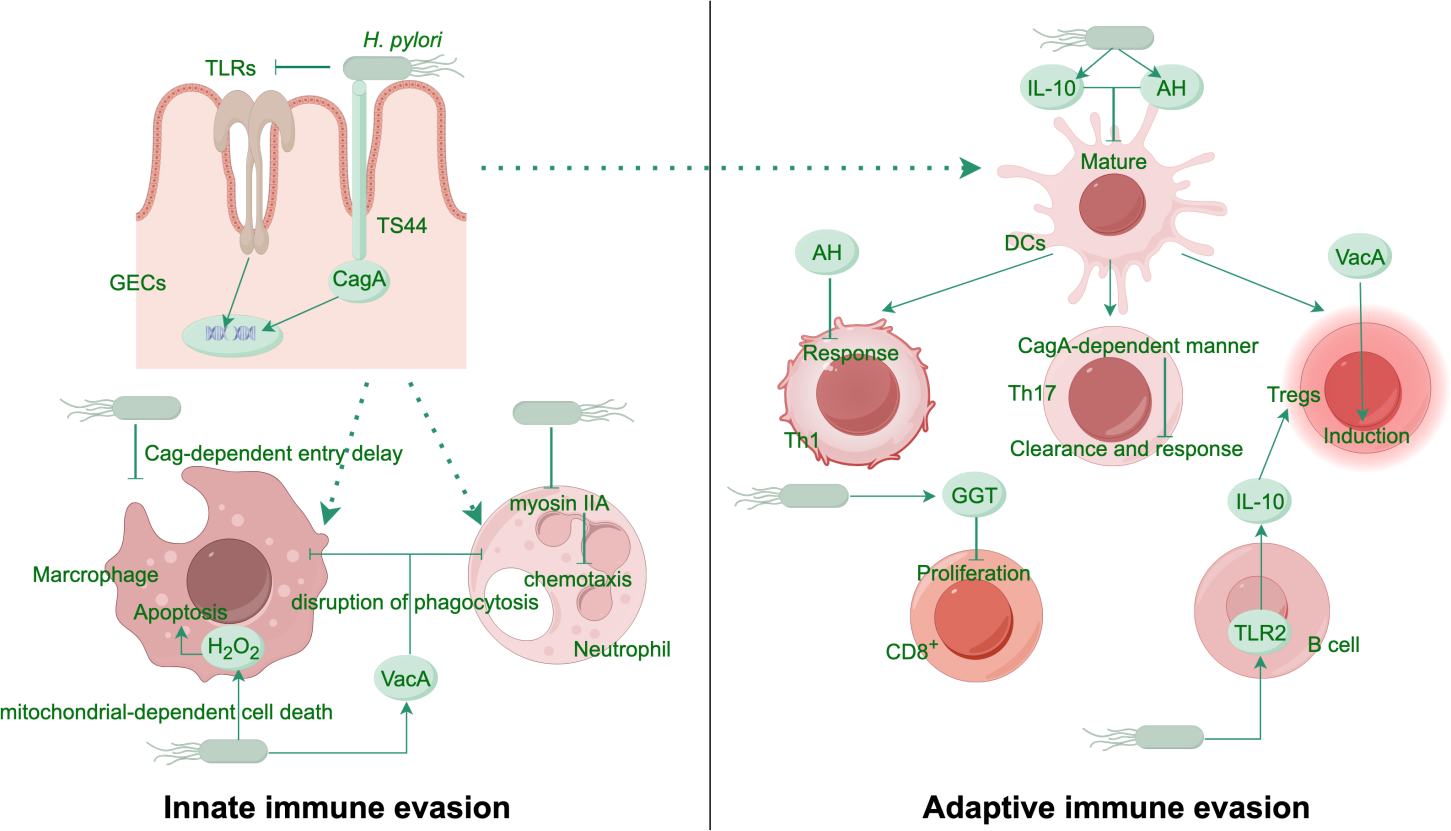
*H. pylori* evasion mechanisms from host innate and adaptive immune responses. *H. pylori* evades immune recognition by modifying its PAMPs and manipulating the antigen-presenting capacity of phagocytes. In a Cag-dependent manner, the bacterium delays entry into macrophages and promotes macrophage apoptosis through H_2_O_2_ production. It inhibits the myosin IIA protein in neutrophils, thereby disrupting their chemotaxis. The virulence factor VacA blocks phagosome-lysosome fusion, resisting phagocytosis by innate immune cells. The bacterial metabolite AH attenuates mature of DCs and suppresses Th1 responses. In a CagA-dependent manner, *H. pylori* also inhibits Th17-mediated clearance. Another virulence factor GGT induces G1 cell cycle arrest in T cells, curbing their proliferation. VacA promotes the expansion of Tregs, while *H. pylori* further enhances the differentiation of immunosuppressive Tregs via the TLR2/MYD88 pathway by stimulating B cells to secrete more IL-10. AH, ADP-heptose; DCs, dendritic cells; GECs, gastric epithelial cells; GGT, g-glutamyl-transpeptidase; PAMPs, pathogen-associated molecular patterns; TLRs, Toll like receptors; Tregs, regulatory T cells.

The immune response to *H. pylori* infection is intricate and involves both innate and adaptive immunity. The body’s innate immune defense recognizes *H. pylori* by utilizing pattern recognition receptors (PRRs), like Toll-like receptors (TLRs), which activate the generation of proinflammatory signaling proteins and chemotactic factors (Nagashima and Yamaoka, 2019). However, *H. pylori* has developed strategies to modulate this response, including the downregulation of TLR signaling and the inhibition of antigen presentation, thereby dampening the immune response and facilitating persistent infection (Bergman et al., 2004). *H. pylori* infection triggers innate immune responses, yet it suppresses immune-cell activation, thereby reducing the efficiency of macrophages and neutrophils in clearing the pathogen (Karkhah et al., 2019; Fuchs et al., 2023). T cell and B cell activation form the core of the adaptive immune response, culminating in the creation of antibodies specific to *H. pylori* antigens (Reyes and Peniche, 2019; Sirit and Peek, 2025) (**Figure 1**). Despite this robust immune response, *H. pylori*’s ability to express antigenic variation and implement strategies to evade immune clearance often result in chronic infection (El-Omar et al., 2000). Infection with *H. pylori* might increase the expression of proliferating cell nuclear antigen (PCNA) while simultaneously decreasing the expression of Bcl-2-associated X protein (Bax). This modulation promotes tumor cell proliferation and inhibits apoptosis (Wang et al., 2015).

## 3 Association of *H. pylori* with nongastric digestive cancers

### 3.1 Hepatocellular carcinoma

#### 3.1.1 Associated evidence

Globally, primary liver cancer represents a major contributor to both cancer incidence and mortality. It ranks as the sixth most common cancer worldwide and the third leading cause of cancer-related deaths (Bray et al., 2024). A pronounced geographic disparity exists in HCC incidence, with high frequency observed across Asia and Africa but low occurrence in Europe and North America. Regardless of regional incidence rates, virtually all areas report male incidence rates that are two- to three-fold higher than those in femaless (Altekruse et al., 2009; Bray et al., 2024). A growing body of epidemiological evidence suggests a potential association between *H. pylori* infection and the onset of HCC. Studies have detected *H. pylori* and related species in liver samples from patients afflicted with HCC and CCA, indicating a possible association (Esmat et al., 2012; Waluga et al., 2015). A single-center observational study identified *H. pylori* as an initiating factor for complications in cirrhotic patients that can potentially increase the incidence of HCC, with the eradication of *H. pylori* infection possibly reducing the incidence of such complications (Abdel-Razik et al., 2020). Furthermore, a comprehensive meta-analysis from 1994–2023 across key literature databases revealed a substantial increase in the risk of HCC associated with *H. pylori* infection, with an overall odds ratio of 4.75 (95% CI, 3.06–7.37). This risk is exacerbated when *H. pylori* infection is combined with hepatitis C virus (HCV) infection (Madala et al., 2021). The existing evidence predominantly linking HP infection to HCC is largely derived from epidemiological studies and the presence of HP at the tumor site.

However, in a nested case–control study evaluating the association between seropositivity for *H. pylori* proteins and the subsequent development of HCC or biliary cancers, no significant association was detected between seropositivity for these 15 proteins and the subsequent incidence of HCC or biliary cancers. Despite the conclusions of this study suggesting a potential possible association, the findings are limited by the study population, which consisted exclusively of white individuals, and assessed only antigens specific to *H. pylori*. Notably, in this study, *H. pylori* seropositivity was not found to be associated with liver cancer. This lack of association remained consistent even upon subgroup analysis of CagA+ strains (Makkar et al., 2020). Similarly, in a mouse model study examining the role of *H. pylori* infection in promoting HCV-associated HCC, it was determined that *H. pylori* does not facilitate the progression of HCC. Furthermore, research has indicated that the presence of the HCV transgene might contribute to improvements in certain liver and gastric lesions observed in mice concurrently infected with *H. pylori* (García et al., 2013). According to meta-analysis results published in the past 20 years, *H. pylori* infection and HCC occurrence are positively correlated, and the correlation is stronger in specific subgroups, including patients with hepatitis B, hepatitis C, cirrhosis, etc. Patient bile and serum samples are particularly useful in the diagnosis of *H. pylori* infection (Wang et al., 2016b; Wang et al., 2016a; Madala et al., 2021; Samragnyi et al., 2021; Penaflorida et al., 2024).

The development of HCC can also be attributed to chronic viral hepatitis and nonalcoholic fatty liver disease (NAFLD). In the context of chronic viral hepatitis, a comprehensive search performed across multiple databases suggests that *H. pylori* infection may significantly influence the progression of chronic hepatitis B virus (HBV) and hepatitis C virus (HCV) infection, as well as its association with HBV-associated cirrhosis and hepatocellular carcinoma (HCC) (Wang et al., 2016b; Wang et al., 2016a).

Furthermore, a wide range of opinions exist regarding the association between the development of NAFLD and *H. pylori*. A recent transcriptomic study in a murine model demonstrated that *H. pylori* infection exacerbates hepatic steatosis associated with metabolic dysfunction by modulating the lipid metabolic pathway, with the *H. pylori* virulence factor CagA playing a crucial role in this regulatory mechanism (Chen et al., 2024). However, a bidirectional Mendelian randomization study using the GWAS database to evaluate the causal relationship between *H. pylori* infection and NAFLD suggests that there is currently insufficient genetic evidence to support a causative association between *H. pylori* infection and NAFLD. This study also indicates that eradicating or preventing *H. pylori* infection may not confer any discernible benefits for individuals with NAFLD (Liu et al., 2022). Conversely, a randomized controlled trial investigating the impact of *H. pylori* eradication on patients with NAFLD revealed that after one year of treatment, metabolic parameters associated with NAFLD were further reduced, and liver steatosis was ameliorated, suggesting that screening individuals at risk for NAFLD for *H. pylori* infection may be advantageous (Yu et al., 2022). A meta-analysis of 34 studies with a total of 175,575 subjects up to January 2024 revealed significantly higher rates of NAFLD among individuals positive for *H. pylori* than among those negative for *H. pylori* in 14 studies. In 17 studies (74,928 cases), the prevalence of *H. pylori* infection was significantly greater in NAFLD patients than in patients without NAFLD, providing compelling evidence supporting a strong correlation between *H. pylori* infection and susceptibility to NAFLD (Zhang et al., 2024). This association has been shown to be significant in specific populations, such as individuals with diabetes and females, on the basis of multiple cross-sectional studies (Wang et al., 2021; Chen et al., 2023). Despite these findings, multiple clinical studies have shown no significant correlation between *H. pylori* infection and the development of HCC. A cross-sectional study in Japan involving 13,737 participants who received medical checkups revealed no significant association between *H. pylori* infection status and the presence of fatty liver disease, including NAFLD (Okushin et al., 2015). A two-year cohort study in China and a study in Korea involving 3663 healthy individuals reported no significant association between *H. pylori* infection and NAFLD (Baeg et al., 2016; Zheng et al., 2025). Similarly, several single-center, cross-sectional studies have shown no significant association between *H. pylori* infection and NAFLD (Cai et al., 2018; Fan et al., 2018).

In summary, the association between *H. pylori* infection and HCC is inconclusive. While some epidemiological studies and meta-analyses suggest a potential link between an increased risk of HCC and infection with *H. pylori*, especially in the context of HCV coinfection, other research, including a nested case–control study and a mouse model, revealed no significant correlation (**Table 2**). A bidirectional Mendelian randomization study also revealed insufficient genetic evidence to support a causative link between *H. pylori* and NAFLD, a condition related to HCC. Despite some studies showing a higher prevalence of NAFLD in *H. pylori*-positive individuals, multiple cross-sectional studies reported no significant association between *H. pylori* infection and NAFLD, emphasizing the need for further research to clarify these relationships.

**Table 2:** Evidence of associations between *H. pylori* and HCC and risk factors for HCC.

Year	Subject	Study design	HCC/Risk factors for HCC	Conclusion	Reference
2008	10 studies, including 242 cases and 280 controls	Meta-analysis	HCC	Positive	(Samragnyi et al., 2021)
2015	13,737 subjects	Cross-sectional study	NAFLD	Negative	(Okushin et al., 2015)
2016	3,663 subjects	Retrospective observational study	NAFLD	Negative	(Baeg et al., 2016)
2016	5 studies, including 163 cases and 544 controls	Meta-analysis	HCC	Positive	(Wang et al., 2016b)
2016	8 studies, 337 cases and 1426 controls	Meta-analysis	HCC	Positive	(Wang et al., 2016a)
2018	21,456 subjects	Cross-sectional study	NAFLD	Negative	(Fan et al., 2018)
2018	2,051 subjects	Retrospective observational study	NAFLD	Negative	(Cai et al., 2018)
2020	558 patients with cirrhosis	Single-center prospective cohort study	HCC	Possible	(Abdel-Razik et al., 2020)
2020	105 Cases and 357 controls	Case–control study	HCC	Negative	(Makkar et al., 2020)
2020	9 *H. pylori*-infected and 19 sham *H. pylori*-uninfected mice	Controlled experimental study	HCC	Negative	(García et al., 2013)
2021	26 studies, including 866 cases and 3585 controls	Meta-analysis	HCC	Positive	(Madala et al., 2021)
2022	1,483 cases and 17,781 controls from GWAS	Bidirectional Mendelian randomization study	HCC	Negative	(Liu et al., 2022)
2022	200 patients with *H. pylori* infection	Randomized controlled trial	NAFLD	Positive	(Yu et al., 2022)
2023	Diabetic group: NAFLD, 3044 cases; Non-NAFLD: 4146 cases	Cross-sectional study	NAFLD	Positive	(Liu et al., 2022)
2024	32 case mice and 16 control mice	Controlled experimental study	MASLD	Positive	(Chen et al., 2024)
2024	34 studies, including 175,575 subjects	Meta-analysis	NAFLD	Positive	(Zhang et al., 2024)
2024	2,063 subjects	Cross-sectional study	NAFLD	Negative	(Zheng et al., 2025)
2024	8 studies, including 1392 cases and 1256 controls	Meta-analysis	HCC	Positive	(Penaflorida et al., 2024)

MASLD, Metabolic dysfunction-associated steatotic liver disease; NAFLD, Nonalcoholic fatty liver disease; HCC, Hepatocellular carcinoma; GWAS, Genome-wide association study.

#### 3.1.2 Possible mechanisms

The potential mechanisms by which *H. pylori* infection may promote HCC are multifaceted and not yet fully understood. On the basis of the available literature, the following mechanisms have been proposed. First, *H. pylori* infection has been shown to promote liver injury through an exosome-mediated mechanism, which may contribute to hepatocellular carcinoma development (Mohammadi Azad et al., 2024). Specifically, *H. pylori* infection activates the NF-κB and PI3K/AKT signaling pathways, both of which are intricately linked to hepatic inflammation and tumorigenesis. This activation enhances the proliferation, migration, and invasion capabilities of HepG2 and Hep3B cells, providing a mechanistic explanation for liver damage caused by *H. pylori* infection (Zhang et al., 2024). Emerging evidence suggests a pathogenic synergy between *H. pylori* colonization and chronic hepatotropic viral infections (HBV/HCV) in HCC development. This could be due to chronic portal hypertension-induced gastric mucosal barrier compromise facilitates bacterial translocation via portosystemic shunting (Madala et al., 2021). Moreover, CagA+ strains induce mitochondrial-dependent apoptosis in HepG2 cells through ROS-mediated p38 MAPK/JNK activation, while concurrently upregulating pro-survival Bcl-xL expression (Mohammadi et al., 2023). Bile duct-ligated murine models reveal *H. pylori’s* capacity to colonize peribiliary glands, triggering TLR4/MyD88-dependent IL-6 production that activates quiescent hepatic stellate cells into collagen-secreting myofibroblasts (Goo et al., 2009; Huang et al., 2009). Furthermore, *H. pylori* lysates impair SMAD2/3 nuclear translocation in LX-2 cells, creating TGF-β1 signaling paradox characterized by simultaneous fibrogenesis promotion and growth inhibition escape (Ki et al., 2010).

In summary, *H. pylori* infection may play a role in the development and progression of HCC through various mechanisms. These include directly infecting liver tissues, interacting with chronic liver diseases, altering the balance between hepatocyte proliferation and apoptosis, and potentially affecting HCC cell proliferation, invasion, and metastasis. These findings necessitate re-evaluation of antimicrobial eradication protocols in cirrhotic populations and development of microbiome-targeted adjuvant therapies.

### 3.2 Biliary tract cancer

#### 3.2.1 Associated evidence

Biliary tract cancer (BTC) is a group of tumors including gallbladder cancer (GBC), extrahepatic cholangiocarcinoma (ECC), and intrahepatic cholangiocarcinoma (ICC).The incidence and mortality of biliary tract cancer (BTC) are rising worldwide, with the sharpest increases observed in South America and Asia. Epidemiologic studies show the disease peaks between ages 50 and 70. Intrahepatic and perihilar cholangiocarcinomas are about 1.5-fold more common in men, whereas gallbladder carcinoma is two- to six-fold more frequent in women (Ghidini et al., 2019). Retrospective studies show higher *H. pylori* detection in cholangiocarcinoma (CCA) patients than in healthy controls (Boonyanugomol et al., 2012; Avilés-Jiménez et al., 2016), with subgroup odds ratios (ORs) of 4.18 (CCA), 1.36 (GBC), and 5.93 (other BTC) (Cherif et al., 2020). The enrichment of *H. pylori* virulence factors (CagA, VacA) in ECC further supports this association (Avilés-Jiménez et al., 2016). Additionally, *H. pylori* has been associated with cholelithiasis and chronic cholecystitis but not gallbladder polyps (Lim et al., 2023). A U.S. national study (2016–2020) confirmed these findings, with chronic cholecystitis exhibiting the highest risk (Ahmad et al., 2024). In the Finnish ATBC study, a positive correlation was observed between seropositivity for *H. pylori* protein and an elevated risk of BTC, with an OR for all BTC combined of 5.47 (95% CI: 1.17–25.65) (Murphy et al., 2014). However, some studies, including a nested case-control analysis, found no significant link, possibly due to population differences or detection methods (Fallone et al., 2003; Makkar et al., 2020).

In a study evaluating the impact of coinfection on hepatobiliary abnormalities in hamsters, concurrent infection with *H. pylori* and *Opisthorchis viverrini* led to the most severe hepatobiliary lesions, characterized by periductal fibrosis, cholangitis, and bile duct hyperplasia (Dangtakot et al., 2017). L-fucose may serve as a viable receptor for *Helicobacter pylori*, facilitating its colonization in the gut environment of *Opisthorchis viverrini* (Penaflorida et al., 2024). Moreover, *O. viverrini*, which acts as a carrier of CagA+ *H. pylori*, comigrates to the bile duct, facilitating the colonization of *H. pylori* and exacerbating both pathogenesis and carcinogenesis in the biliary duct (Suyapoh et al., 2021).

Most evidence supports a *H. pylori*-BTC association, but inconsistencies highlight the need for further research to clarify mechanisms and guide clinical strategies (**Table 3**).

**Table 3:** Evidence of an association between *H. pylori* infection and BTC.

Year	Country	Subject/Patients	Study design	Conclusion	Reference
2003	Canada	75 biliary stones, 15 pancreatic-biliary malignancies and 4 primary sclerosing cholangitis	Case–control study	Negative	(Fallone et al., 2003)
2012	Thailand	140 subjects	Cross-sectional study	Positive	(Boonyanugomol et al., 2012)
2014	USA	64 biliary cancers, 122 liver cancers, and 224 age-matched controls	Case–control study	Positive	(Murphy et al., 2014)
2016	Mexico	200 subjects	Cross-sectional study	Positive	(Avilés-Jiménez et al., 2016)
2020	All	473 cancers and 596 controls	Meta-Analysis	Positive	(Cherif et al., 2020)
2020	USA	74 biliary cancers, 105 liver cancers, and 357 matched controls	Case–control study	Negative	(Makkar et al., 2020)
2024	USA	32,966,720 subjects	Cross-sectional study	Positive	(Ahmad et al., 2024)

#### 3.2.2 Possible mechanisms

*H. pylori* contributes to biliary tract cancer (BTC) through multiple interconnected mechanisms. Chronic inflammation driven by persistent infection creates a pro-carcinogenic microenvironment, promoting genomic instability when combined with other carcinogens (Mishra et al., 2019; Dangtakot et al., 2021). The bacterium may colonize biliary tissues via duodenobiliary reflux (Krasinskas et al., 2007), with clinical studies linking its presence to gallbladder cancer and cholangiocarcinoma (CCA) development, potentially through stone formation (Cherif et al., 2019; Grigor’eva and Romanova, 2020) or direct epithelial transformation (Thanaphongdecha et al., 2020). Specifically, this study investigated the impact of *H. pylori*-GGT on the induction of apoptosis and the stimulation of IL-8 production in a human bile duct cancer cell line (KKU-100) (Boonyanugomol et al., 2012). Key virulence factors like GGT induce IL-8 production and apoptotic dysregulation in bile duct cells, while CagA+ strains modulate proliferation and inflammatory responses in CCA (Boonyanugomol et al., 2011; Boonyanugomol et al., 2013). Emerging evidence indicates that *H. pylori* infection exerts significant oncogenic effects in BTC. *In vitro* studies using human cholangiocarcinoma models have revealed that *H. pylori* infection induces the activation of NF-κB signaling pathway, which subsequently stimulates VEGF upregulation and facilitates tumor-associated angiogenesis. This pathogenic cascade may substantially accelerate the progression of cholangiocellular carcinoma through enhanced vascularization (Takayama et al., 2010). Notably, the bacterium’s virulence factors appear to participate in the pathogenesis of GBC. Experimental evidence indicates that gallbladder-derived *H. pylori* strains exhibit marked cytotoxic potential against primary gallbladder epithelium in cell culture systems, initiating a characteristic sequence of cellular degeneration progressing through cytoplasmic vacuolization, membrane disintegration, necrotic changes, and ultimately culminating in programmed cell death (Zhao et al., 2024).

### 3.3 Esophageal carcinoma

#### 3.3.1 Associated evidence

Globally, esophageal cancer ranks as the seventh most frequently diagnosed malignancy and the sixth leading cause of cancer death, accounting for an estimated 604–000 new cases and 544–000 deaths in 2020. The burden is markedly skewed toward men, who represent roughly 70% of diagnoses and experience incidence and mortality rates two- to three-fold higher than women (Obermannová et al., 2022). Oesophageal cancer is broadly classified into two clinicopathologically distinct entities: esophageal squamous-cell carcinoma (OSCC) and esophageal adenocarcinoma (OAC). OSCC dominates the global landscape, representing approximately 90% of all cases and showing pronounced geographic clustering in East Asia, East Africa and South America (Smyth et al., 2017). Recent research has explored the complex relationship between H. pylori infection and EC, revealing both protective and promotional effects. Esophageal carcinoma (EC) is a significant global health concern originating from the esophageal epithelium, with four main subtypes: esophageal squamous cell carcinoma (ESCC), esophageal adenocarcinoma (EAC), undifferentiated carcinoma, and rarer forms. ESCC and EAC account for over 95% of cases. Recent research has explored the complex relationship between *H. pylori* infection and EC, revealing both protective and promotional effects.

Epidemiological and molecular studies suggest that chronic *H. pylori* infection, particularly with CagA-positive strains, may protect against esophageal carcinogenesis through immunomodulation. This contrasts with other findings indicating that *H. pylori* either promotes cancer or has no significant impact. A recent study reported a significantly lower rate of *H. pylori* infection (4.5%) in patients with EC than in the general population in a cohort from Spain (López-Gómez et al., 2024). This finding challenges the established negative correlation between *H. pylori* and gastric malignancies and suggests that *H. pylori* may have a potential protective effect against EC, especially considering the widespread use of proton pump inhibitors (Omer and Habtemariam, 2024).

Meta-analyses consistently demonstrate H. pylori’s inverse association with EAC and Barrett’s esophagus (BE), attributed to reduced gastric acid secretion and GERD mitigation (Rokkas et al., 2007; Permuth et al., 2018). Via a subgroup analysis, Xie et al. also reported there was no correlation between HP and ESCC in the overall population (Xie et al., 2013). Similarly, colonization by *H. pylori*, particularly CagA+ strains, was found to be negatively correlated with the incidence of BE. This negative correlation may be mediated through the effects of gastroesophageal reflux disease (GERD) (Wang et al., 2018). Furthermore, Nie demonstrated a negative association between CagA+ HP colonization and ESCC in Asian populations (Nie et al., 2014). A summary of 5 meta-analyses of the occurrence factors of EAC and ESCC, which is consistent with most of the previous results, revealed that *H. pylori* may play a protective role in EAC and has no correlation with ESCC (Castro et al., 2018). These developments seem to suggest a potential protective association between *H. pylori* infection and ESCC in Asian and Middle East populations (Gao et al., 2019). A review of the association between *H. pylori* and EC in Asian populations revealed that there was no significant association between *H. pylori* and EC in Asian populations but that there was significant heterogeneity across studies, possibly due to differences in population characteristics, the number of cases and controls, *H. pylori* testing methods, and overall study design. Thus, the “protective” nature of this association may be overestimated in Asian populations (Liu and Liu, 2024).

**Figure 2:**
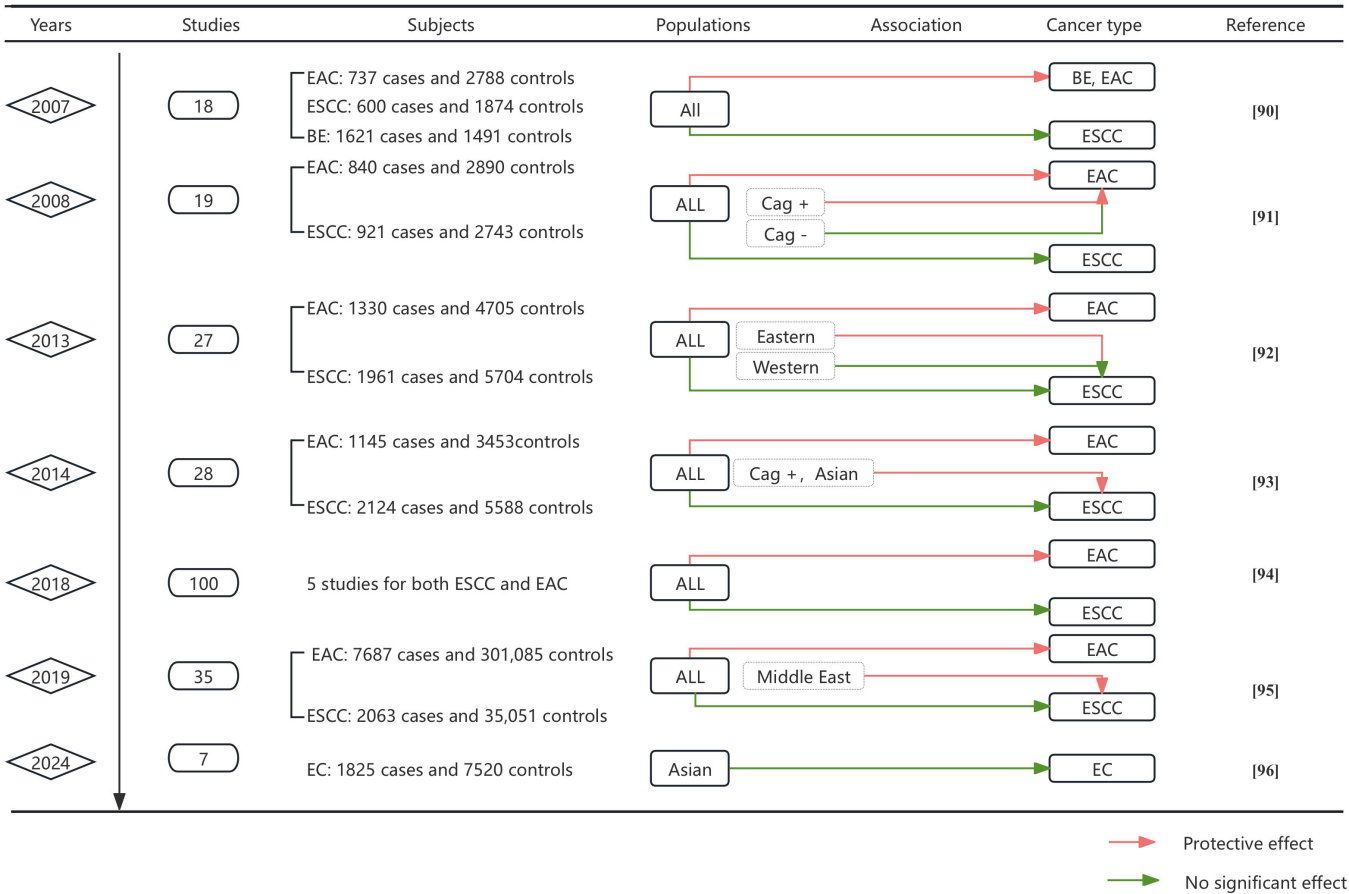
Meta-analysis by time progression of the association between *H. pylori* and EC. This figure synthesizes key findings from 11 meta-analyses evaluating *H. pylori* infection and esophageal cancer risk. Data include publication year, number of studies analyzed, subject counts (cases/controls), population characteristics, association direction, cancer subtypes, and references. Color-coded association indicators: Green lines denote statistically significant protective effects of *H. pylori* infection against cancer development, Red lines indicate no significant association between infection and cancer risk.

In summary, the association between *H. pylori* and EC appears to be subspecies specific (**Figure 2**). The majority of evidence supports a protective effect of *H. pylori* in EAC, whereas its association with ESCC is less pronounced. Stratified analysis suggested that the link between *H. pylori* and ESCC may be overestimated in Eastern populations, contributing to the high heterogeneity observed across studies. Further research is warranted to elucidate the relationship between *H. pylori* and ESCC (Omer and Habtemariam, 2024).

#### 3.3.2 Possible mechanisms

The relationship between *H. pylori* and esophageal cancer (EC) remains complex. While many studies highlight a protective effect—linked to reduced gastric acid secretion (via atrophy or PPIs) and lower GERD risk (Maity et al., 2024; Zhang et al., 2024)—some suggest *H. pylori* may promote Barrett’s esophagus (BE) and esophageal adenocarcinoma (EAC) through COX-2 upregulation via microRNAs (Kountouras et al., 2012; Teng et al., 2018).

In the specific context of BE, *H. pylori* infection has the potential to induce apoptosis in gastric adenocarcinoma cells that are evolving from BE. This process is mediated through the Fas apoptotic pathway, thereby curbing the progression of these cells into EC. Moreover, *H. pylori* infection can influence the synthesis of ghrelin, a hormone that plays a key role in regulating appetite and energy balance. Alterations in ghrelin levels may indirectly affect obesity and GERD, both of which are established risk factors for EC (Murphy et al., 2012).

### 3.4 Colorectal cancer

#### 3.4.1 Associated evidence

CRC ranks as the third most frequently diagnosed malignancy and the second leading cause of cancer-related death worldwide, with its incidence and mortality rates steadily rising (Abedizadeh et al., 2024). Arising from neoplastic transformation of colonic glandular epithelium, CRC develops exclusively in the colon or rectum and is broadly categorized into three major forms: sporadic, hereditary, and colitis-associated (Hossain et al., 2022). The relationship between *H. pylori* infection and colorectal cancer (CRC) remains complex and controversial. While CRC is a leading cause of cancer mortality worldwide with multifactorial etiology, numerous studies have investigated *H. pylori*’s potential role with conflicting results. Several lines of evidence suggest a positive association, particularly seropositivity for virulence factors like HcpC and VacA which correlate with increased CRC risk (Butt et al., 2020). Meta-analyses demonstrate stronger associations in East Asian populations and links with colorectal adenomas and advanced lesions (Choi et al., 2020; Ma et al., 2023). The link between *H. pylori* infection and an increased risk of CRC is further supported by the majority of published studies, which suggest that chronic atrophic gastritis, induced by *H. pylori*, enhances susceptibility to colorectal tumors and that the presence of *H. pylori* infection in individuals with metabolic syndrome further increases this risk (Lin et al., 2010; Kountouras et al., 2017; Venerito et al., 2017). Moreover, the role of *H. pylori* in the development of CRC is evident in studies examining *H. pylori*-related metabolic syndrome, which may precipitate the formation of digestive tract tumors. In specific subpopulations, such as those with elevated glycated hemoglobin or NAFLD, the eradication of *H. pylori* has shown potential in curbing these carcinogenic properties (Kountouras et al., 2018). Importantly, however, the susceptibility to the association between *H. pylori* and CRC may differ among various ethnic groups (Blase et al., 2016; Butt and Epplein, 2019; Butt et al., 2019). A study examining the risk of colorectal adenoma (CRA) following *H. pylori* eradication in a Swedish national population revealed that overall *H. pylori* eradication was associated with an increased risk of CRA. The risk of CRA was elevated 1–2 years after eradication but decreased to below baseline levels at 2–4 years and remained low at 4–6 years. Overall, *H. pylori* eradication was not consistently associated with a significant reduction in CRC incidence in the Swedish cohort (Liu et al., 2024). A comparable association was observed in the Chinese population, where the initial incidence of CRC was notably higher among individuals infected with *H. pylori* and subsequently declined to levels below those of the general population, particularly for rectal cancer, approximately 10 years after successful eradication of *H. pylori* (Guo et al., 2023). The cooccurrence of CRC with *H. pylori* infection has been extensively documented in numerous studies. Although the specific clinical benefits of *H. pylori* eradication therapy have yet to be conclusively established, the simplicity of detecting and treating *H. pylori* suggests that eradication may still represent a potentially beneficial intervention (Wernly et al., 2023). A retrospective cohort study conducted among veterans in the United States revealed that *H. pylori* positivity was significantly associated with increased CRC incidence and mortality. Over a 15-year follow-up period, individuals who received *H. pylori* eradication therapy presented a lower CRC incidence and mortality than those who did not receive treatment. Specifically, untreated *H. pylori*-infected individuals had CRC incidence and mortality rates that were 23% and 40% higher, respectively, than those of *H. pylori*-infected individuals who underwent eradication therapy. These findings provide further evidence that *H. pylori* eradication therapy may be beneficial in preventing CRC (Shah et al., 2024).

However, significant limitations and contradictory evidence temper these conclusions. No association was found in elderly Caucasian populations (Blase et al., 2016), and Mendelian randomization analysis failed to establish genetic evidence for causality (Luo et al., 2023). This lack of a genetically - established association, despite some observational correlations, underscores the intricate and potentially variable nature of *H. pylori’s* role in CRC.

The presence of *H. pylori* has been identified in both CRA and CRC tissues (Kapetanakis et al., 2012), hinting at a possible biological interaction. However, as reported in a European Prospective Investigation into Cancer and Nutrition (EPIC) cohort, the biological mechanisms that potentially underlie the causal role of *H. pylori* in the development of CRC remain unclear. Moreover, it is uncertain whether subsequent eradication of *H. pylori* can effectively reduce CRC incidence (Butt et al., 2020). This uncertainty emphasizes the necessity for further research to elucidate the relationship between *H. pylori* infection and colorectal neoplasm development, as the existing evidence does not consistently support a direct causal connection (**Table 4**).

**Table 4:** Evidence linking *H. pylori* to CRC.

Year	Country	Subject/Patients	Study design	Conclusion	Reference
2010	China	9311 subjects	Cross-sectional study	Positive	(Lin et al., 2010)
2016	Caucasian	392 cancer cases and 774 controls	Case–control study	Negative in elderly, mostly Caucasian population	(Blase et al., 2016)
2019	USA	4063 cancer cases and 4063 matched controls	Case–control study	Positive	(Butt et al., 2019)
2020	European	485 colorectal cancer cases and 485 matched controls	Case–control study	Positive	(Butt et al., 2020)
2020	All	48 studies including 171,045 subjects	Meta-analysis	Positive	(Choi et al., 2020)
2023	East Asian	9 studies including 6355 subjects	Meta-analysis	Positive in China/Negative in Japan and Korea	(Ma et al., 2023)
2023	ALL	/	Bidirectional Mendelian randomization study	Negative	(Liu et al., 2022)
2023	Austria	5,707 subjects	Cross-sectional study	Positive	(Wernly et al., 2023)
2024	Swedish	80,381 subjects	Population-based cohort study	Positive	(Liu et al., 2024)
2024	China	96,572 subjects	Population-based cohort study.	Positive	(Guo et al., 2023)
2024	USA	812,736 subjects	Population-based cohort study	Positive	(Shah et al., 2024)

#### 3.4.2 Possible mechanisms

*H. pylori* contributes to colorectal carcinogenesis through multiple interconnected mechanisms. Recent studies have elucidated several key pathways. Direct interaction with colonic mucosa is evidenced by bacterial detection in polyps (Soylu et al., 2008; Li et al., 2025). The infection dysregulates critical cellular processes by increasing proliferation (elevated Ki-67) and inhibiting apoptosis (reduced Bax/elevated Bcl-2*)* (Kapetanakis et al., 2012; Kountouras et al., 2017), while also activating cancer stem cells and inducing oncogenes (Kountouras et al., 2017).

The bacterium significantly alters gut microbiota homeostasis, promoting gastrin release that stimulates colorectal cell growth (Fireman et al., 2000; Georgopoulos et al., 2006). Moreover, infection may contribute to cancer progression by disrupting the homeostasis of the gut microbiota, an essential factor in maintaining intestinal health (Kienesberger et al., 2016; Dash et al., 2019). Concurrently, H. pylori triggers chronic inflammation through inflammatory factor release (Jackson et al., 2009; Tuomisto et al., 2019), establishing a tumor-favorable microenvironment. Furthermore, *H. pylori* infection alters the gut microbiota, influencing intestinal immunity and potentially increasing the risk of CRC (Engelsberger et al., 2024). Key immunomodulatory effects include reduction of regulatory T cells and activation of pro-carcinogenic STAT3 signaling pathways, leading to goblet cell depletion (Ralser et al., 2023). These changes collectively promote epithelial transformation and tumor progression.

### 3.5 Pancreatic cancer

Pancreatic ductal adenocarcinoma (PDAC) has already eclipsed breast cancer as the third deadliest malignancy in the United States, and current trajectories suggest it will surpass colorectal cancer before 2040 to rank second only to lung cancer in cancer-related mortality (Halbrook et al., 2023). In the U.S., the disease is typically diagnosed at a median age of 71 years, with incidence modestly higher in men than in women (5.5 versus 4.0 cases per 100,000) (Park et al., 2021). Emerging evidence suggests H. pylori may contribute to pancreatic carcinogenesis, though findings remain inconsistent. While some serological studies demonstrate positive associations (Raderer et al., 1998; Risch et al., 2010; Osaki et al., 2022). However, it is important to acknowledge that the relationship is not consistent, as other large cohort studies have shown either no correlation or an inverse correlation between *H. pylori* infection and pancreatic cancer (de Martel et al., 2008; Hirabayashi et al., 2019; Permuth et al., 2021). This highlighting the complexity of *H. pylori’s* oncogenic potential.

Mechanistically, H. pylori infection promotes secretion of proinflammatory cytokines (IL-8, VEGF) and activates oncogenic pathways (NF-κB) in pancreatic cells (Chen et al., 2025), providing biological plausibility for epidemiological observations. These findings underscore the need for further investigation into H. pylori’s role in pancreatic cancer development.

## 4 Limitations and future directions

Current evidence for *H. pylori’s* role in extragastric malignancies remains limited by methodological inconsistencies, including geographic/ethnic variability in study populations, divergent diagnostic approaches (serological *vs* molecular), and inadequate control for confounders like smoking or viral coinfections. These limitations, compounded by heterogeneous study designs, hinder reliable extrapolation of findings. Mechanistic insights are particularly constrained by the lack of translational validation through appropriate animal models or 3D organoid systems that could better recapitulate human tumorigenesis. Moving forward, resolving these uncertainties will require large-scale multicenter cohorts employing standardized diagnostic protocols, coupled with advanced molecular profiling to elucidate strain-specific effects. Simultaneously, genetically engineered models must be developed to validate proposed oncogenic pathways, while interdisciplinary collaboration will be essential for comprehensively characterizing host-pathogen interactions. Such integrated approaches promise to clarify *H. pylori’s* potential etiological contributions while identifying clinically relevant biomarkers and therapeutic targets for extragastric cancers associated with this persistent infection.

## 5 Conclusion

Our review demonstrates that H. pylori exhibits distinct associations with extragastric digestive cancers, extending beyond its established gastric carcinogenicity. Compelling epidemiological and mechanistic evidence supports a significant association with increased risk of biliary tract cancers (particularly cholangiocarcinoma, OR 4.18) and, to a more variable extent, hepatocellular carcinoma (OR 4.75, especially with HCV coinfection). In contrast, H. pylori (notably CagA+ strains) confers a protective effect against esophageal adenocarcinoma. Associations with colorectal cancer show population-specific patterns (increased risk in East Asia), while links to pancreatic cancer remain inconsistent. Proposed oncogenic mechanisms involve chronic inflammation, direct virulence factor activity (CagA/VacA), and microbiome dysbiosis. These findings highlight the potential for targeted therapeutic strategies, including eradication in high-risk populations.
